# Age-Dependent Relationship Between Plasma Aβ40 and Aβ42 and Total Tau Levels in Cognitively Normal Subjects

**DOI:** 10.3389/fnagi.2019.00222

**Published:** 2019-09-03

**Authors:** Lih-Fen Lue, Ming-Chyi Pai, Ta-Fu Chen, Chaur-Jong Hu, Li-Kai Huang, Wei-Che Lin, Chau-Chung Wu, Jian-Shing Jeng, Kaj Blennow, Marwan N. Sabbagh, Sui-Hing Yan, Pei-Ning Wang, Shieh-Yueh Yang, Hiroyuki Hatsuta, Satoru Morimoto, Akitoshi Takeda, Yoshiaki Itoh, Jun Liu, Haiqun Xie, Ming-Jang Chiu

**Affiliations:** ^1^Civin Neuropathology Laboratory, Banner Sun Health Research Institute, Sun City, AZ, United States; ^2^Division of Behavioral Neurology, Department of Neurology, National Cheng Kung University Hospital, College of Medicine, National Cheng Kung University, Tainan, Taiwan; ^3^Department of Neurology, National Taiwan University Hospital, College of Medicine, National Taiwan University, Taipei, Taiwan; ^4^Department of Neurology, Taipei Medical University, Taipei, Taiwan; ^5^Department of Neurology, Shuang Ho Hospital, Taipei Medical University, New Taipei City, Taiwan; ^6^Department of Neurology, Kaohsiung Chang Gung Memorial Hospital, Kaohsiung, Taiwan; ^7^Department of Internal Medicine, National Taiwan University Hospital, College of Medicine, National Taiwan University, Taipei, Taiwan; ^8^Clinical Neurochemistry Laboratory, Sahlgrenska University Hospital, Mölndal, Sweden; ^9^Department of Psychiatry and Neurochemistry, Institute of Neuroscience and Physiology, University of Gothenburg, Mölndal, Sweden; ^10^Lou Ruvo Center for Brain Health, Cleveland Clinic Nevada, Las Vegas, NV, United States; ^11^Department of Neurology, Renai Branch, Taipei City Hospital, Taipei, Taiwan; ^12^Department of Neurology, National Yang-Ming University, Taipei, Taiwan; ^13^Department of Neurology, Taipei Veterans General Hospital, Taipei, Taiwan; ^14^MagQu Company Limited, New Taipei City, Taiwan; ^15^MagQu LLC, Surprise, AZ, United States; ^16^Hatsuta Neurology Clinic, Osaka, Japan; ^17^Department of Neurology, Osaka City University Graduate School of Medicine, Osaka, Japan; ^18^Department of Physiology, School of Medicine, Keio University, Tokyo, Japan; ^19^Departemnt of Neurology, Sun Yat-Sen Memorial Hospital, Sun Yat-Sen University, Guangzhou, China; ^20^Department of Neurology, Foshan Hospital of Sun Yat-Sen University, Foshan, China

**Keywords:** Alzheimer, plasma, amyloid, tau, immunomagnetic reduction, cognitively normal subjects

## Abstract

Both amyloid plaques and neurofibrillary tangles are pathological hallmarks in the brains of patients with Alzheimer’s disease (AD). However, the constituents of these hallmarks, amyloid beta (Aβ) 40, Aβ42, and total Tau (t-Tau), have been detected in the blood of cognitively normal subjects by using an immunomagnetic reduction (IMR) assay. Whether these levels are age-dependent is not known, and their interrelation remains undefined. We determined the levels of these biomarkers in cognitively normal subjects of different age groups. A total of 391 cognitively normal subjects aged 23–91 were enrolled from hospitals in Asia, Europe, and North America. Healthy cognition was evaluated by NIA-AA guidelines to exclude subjects with mild cognitive impairment (MCI) and AD and by cognitive assessment using the Mini Mental State Examination and Clinical Dementia Rating (CDR). We examined the effect of age on plasma levels of Aβ40, Aβ42, and t-Tau and the relationship between these biomarkers during aging. Additionally, we explored age-related reference intervals for each biomarker. Plasma t-Tau and Aβ42 levels had modest but significant correlations with chronological age (*r* = 0.127, *p* = 0.0120 for t-Tau; *r* = −0.126, *p* = 0.0128 for Aβ42), ranging from ages 23 to 91. Significant positive correlations were detected between Aβ42 and t-Tau in the groups aged 50 years and older, with Rho values ranging from 0.249 to 0.474. Significant negative correlations were detected between Aβ40 and t-Tau from age 40 to 91 (*r* ranged from −0.293 to −0.582) and between Aβ40 and Aβ42 in the age groups of 30–39 (*r* = −0.562, *p* = 0.0235), 50–59 (*r* = −0.261, *p* = 0.0142), 60–69 (*r* = −0.303, *p* = 0.0004), and 80–91 (*r* = 0.459, *p* = 0.0083). We also provided age-related reference intervals for each biomarker. In this multicenter study, age had weak but significant effects on the levels of Aβ42 and t-Tau in plasma. However, the age group defined by decade revealed the emergence of a relationship between Aβ40, Aβ42, and t-Tau in the 6th and 7th decades. Validation of our findings in a large-scale and longitudinal study is warranted.

## Introduction

The greatest risk factor for developing late-onset Alzheimer’s disease (AD) is age. At age 65, the incidence of AD is 3%, and every 5–6 years, the incidence rate doubles (Kukull et al., 2002; Ziegler-Graham et al., 2008). As the population of the world is living longer, it is estimated that there will be over 100 million people with AD by 2050 (Alzheimer’s Association, 2013). To meet the challenges ahead, the development of effective disease-modifying therapeutics and preventative strategies is being accelerated. In the meantime, there is also a pressing need for developing biomarkers whose intended use is for identifying preclinical AD or subjects at risk of AD for clinical trials. Studies have shown that by combining various biomarkers, such as amyloid positron emission tomography (PET), fluorodeoxyglucose (FDG)-PET, magnetic resonance imaging (MRI), and cerebral spinal fluid (CSF) measures of amyloid beta (Aβ), total Tau (t-Tau), and phosphorylated Tau (p-Tau), the accuracy for identifying preclinical AD could be improved (Dubois et al., 2014, 2016; Jack et al., 2016, 2018).

Currently, there are no blood-based biomarkers for identifying the preclinical stage of AD. The progress in developing blood-based biomarkers has been hampered previously by the lack of sensitivity and other technical limitations (Blennow, 2017; Lue et al., 2017a). The most widely used immunoassays for measuring AD biomarkers in CSF produced discordant findings when used in blood (Fei et al., 2011; Olsson et al., 2016; Lövheim et al., 2017; Hanon et al., 2018). Recently, several new technologies have offered superior detection sensitivity and accuracy in measuring blood-based biomarkers (Andreasson et al., 2016; Zetterberg and Blennow, 2018). These innovative technologies include immunomagnetic reduction (IMR) assay (Chiu et al., 2012), single-molecule assay (SIMOA; Janelidze et al., 2016), immuno-infrared-sensor assay (Nabers et al., 2018), and immunoprecipitation-mass spectrophotometry (Nakamura et al., 2018).

Some of these technologies are being validated for classifying mild cognitive impairment (MCI) and AD and for prescreening for PET scans (Chiu et al., 2012, 2013; Tzen et al., 2014; Lue et al., 2017b; Verberk et al., 2018). AD pathological formation precedes the clinical symptoms by one to two decades (Jack et al., 2013). To identify the earliest changes in the blood to reflect the brain pathological state, it is necessary to first establish the normal ranges of the biomarkers Aβ40, Aβ42, and t-Tau. Therefore, in this study, we characterized the age-associated levels of these biomarkers and their relationship within different age groups and finally provided age-related reference intervals for each biomarker. To generalize our research results, this study was conducted in young, middle-aged and older adults with normal cognition across various countries. It is worth noting that this study is cross-sectional, not longitudinal.

## Materials and Methods

### Participating Sites

A total of 391 cognitively normal subjects aged 23–91 were enrolled from 2010 to 2018 from the following six hospitals in Taiwan: National Taiwan University Hospital (NTUH), Taipei Medical University Shuang-Ho Hospital (SHH), Renai Branch of Taipei City Hospital (RAH), Taipei Veterans General Hospital (TVGH), National Cheng Kung University Hospital (NCKUH), and Kaohsiung Chang Gung Memorial Hospital (KCGMH); Sahlgrenska University Hospital (SUH) in Guttenberg, Sweden; Banner Sun Health Research Institute (BSHRI) in Sun City, AZ, USA; two hospitals in the cities of Foshan, Foshan Hospital (FH) and Guangzhou, Sun Yat-Sen Memorial Hospital (SYSMH), Guangdong, China; and finally two hospitals in Japan: Hatsuta Neurology Clinic (HNC) in Osaka, and Osaka City University Hospital (OCUH) in Osaka. All participants were older than 21 years of age and gave their own written informed consent. The study was approved by the Institutional Review Board (IRB) or Research Ethics Committee (REC) of each participating hospital in the respective countries, namely, NTUH REC, Taipei Medical University-Joint IRB for SHH, Taipei City Hospital REC for RAH, TVGH IRB, NCKUH IRB, KCGMH IRB, Central Ethical Review Board-University of Gothenburg for SUH, Banner Health IRB for BSHRI, Sun Yat-Sen University Hospital (SYSUH) Cancer Center IRB, Asai Dermatology Clinic IRB and Osaka City University IRB.

### Cognition Assessment and Criteria for Recruitment

The purpose of the recruitment criteria was to exclude subjects with diagnoses of MCI and dementia. All study sites followed the NIA-AA criteria for the diagnosis of dementia and MCI due to AD (Albert et al., 2011; McKhann et al., 2011). In addition to clinical criteria, basic cognitive assessment tools [Mini-Mental State Examination (MMSE) and Clinical Dementia Rating (CDR)] were also used. The criteria for normal cognition were MMSE ≥ 28 and CDR = 0. Brain imaging and CSF biomarkers were used as supplementary tools. Brain (FDG)-PET were used by HNC/OCUH, Japan, and Subjects from SUH, Sweden had CSF Aβ > 530 pg/ml and t-Tau < 350 pg/ml (Sutphen et al., 2015; Teunissen et al., 2018). Subjects who had acute or chronic systemic diseases or neuropsychiatric disorders, visual or auditory dysfunction severe enough to interfere with cognitive assessments were all excluded.

Numbers of subjects and age profiles of participating hospitals are shown in Table 1.

**Table 1:** The means and standard deviations (SD) of age (years) of the normal-cognition subjects in each participating site*

Site No.	Name of Sites	Subject No.	Median (years)	Minimum (years)	Maximum (years)	Age, years Mean ± SD
1	NTUH-1	90	57	23	81	53.60 ± 17.91
2	NTUH-2	79	69	56	89	70.35 ± 8.12
3	NTUH-3	24	46	26	89	49.54 ± 18.41
4	NCKUH	48	54	33	70	54.15 ± 7.49
5	SHH	38	65	56	76	64.97 ± 5.63
6	KCGMH	27	60	50	72	61.15 ± 4.93
7	SU	18	71	53	89	70.50 ± 9.60
8	BSHRI	16	82	71	91	81.94 ± 5.99
9	RAH	11	64	58	74	64.91 ± 5.05
10	TVGH	17	60	54	88	64.06 ± 10.23
11	SYSH	9	66	45	79	62.67 ± 10.25
12	HNC/OCUH	14	65	53	83	64.93 ± 7.21

*Normal cognition: CDR = 0, MMSE: 28-30, and meet NIA-AA guidelines published in 2011. Name of sites: NTUH, National Taiwan University Hospital; NCKUH, National Cheng Kung University Hospital; SHH, Shuang Ho Hospital; KCGMH, Kaohsiung Chang Gung Memorial Hospital; SUH, Sahlgrenska University Hospital; BSHRI, Banner Sun Health Research Institute; RAH, Renai Branch Taipei City Hospital; TVGHL, Taipei Veterans General Hospital; SYSMH, Sun Yet-Sen Memory Hospital; FH, Foshan Hospital; HNC, Hatsuta Neurology Clinic; OCUH, Osaka City University; SD, standard deviation.

### Preparation of Plasma Samples

Nonfasting plasma samples were collected in EDTA-coated vacutainers (Becton Dickinson, New Jersey, NJ, USA) followed by centrifugation at speeds ranging from 1,500 g to 2,500 g for 15 min at room temperature. The upper layer (plasma) was transferred to a new 15-ml tube, aliquoted into 1.5 ml tubes, and stored at −70°C or lower. Sample aliquots were shipped on dry ice to MagQu Company Limited for IMR assays of Aβ1–40, Aβ1–42 and t-Tau. Assays were performed without knowing the demographic features of the subjects.

### Assays of Aβ40, Aβ42 and t-Tau in Human Plasma

Before the assays, frozen aliquoted samples were thawed on ice. Sample preparation and assays were performed at room temperature. Assays were performed in duplicate for each sample for Aβ40, Aβ42 and t-Tau. The volumes of the reagents and plasma samples were 80 μl reagent (MF-AB0–0060, MagQu) and 40 μl plasma for the Aβ40 assay, 60 μl reagent (MF-AB2–0060, MagQu) and 60 μl plasma for the Aβ42 assay, and 80 μl reagent (MF-TAU-0060, MagQu) and 40 μl plasma for the t-Tau assay. Samples and reagents were mixed briefly in special-sized glass tubes and sealed. The tubes were then placed inside the sample channels of the IMR analyzer (XacPro-S, MagQu) for assay. The concentrations of Aβ40, Aβ42, and t-Tau were calculated according to the standard curves respective to each biomarker. The means of the values obtained from duplicate measurements were calculated for each biomarker and each sample.

### Statistical Analysis

Statistical analysis was performed with MedCalc statistical software version 17.4.4^1^. The statistical significance was defined as *p* < 0.05. Continuous variables according to age groups were analyzed by one-way analysis of variance followed by pairwise differences using the *Student-Newman-Keuls test*. Spearman’s correlations were performed to determine the correlation between the levels of each plasma biomarker and demographic features such as age, sex, and the presence of the ApoEɛ4 allele. The relationship between different biomarkers was also analyzed according to the age groups defined by the intervals of 10 years starting from 20 years of age. Three participants aged 91 were combined into a group of ages 80–89. Multiple stepwise regression analysis was used to determine whether age, ApoE ɛ4 allele, and biomarkers (Aβ40, Aβ42, and t-Tau) had a significant contribution to the levels of a particular biomarker. MedCalc software was used for the construction of age-related reference intervals, and the values of the biomarkers at the 2.5th and 97.5th percentiles were tabulated.

## Results

### Cohort Characteristics

**Figure 1:**
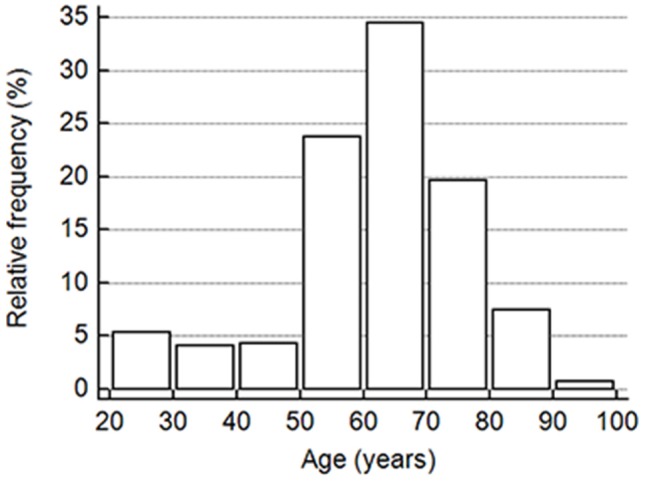
The vertical bar on the y-axis shows the frequency as the percentage in the number of all participants in the cohort, and the x-axis indicates the age groups in 10-year intervals.

This study included a total of 391 cognitively normal subjects aged 23–91 years from 12 participating hospitals. The relative frequency of age distribution is shown in Figure 1. The ApoE genotype information is shown in Table 2. We then determined whether carrying one or two ApoE ɛ4 alleles had effects on any of the biomarker levels. The results in Table 3 show that the only genotype effect was on Aβ40. The Apo-ɛ4 carriers had significantly lower levels of Aβ40.

**Table 2:** The numbers of subjects according to ApoE genotypes.

ApoE genotypes	Number (%)
2/3	19 (11.9)
2/4	3 (1.9)
3/3	110 (68.8)
3/4	27 (16.9)
4/4	1 (0.6)

**Table 3:** The levels of Aβ40, Aβ42, and t-Tau in pg/ml grouped by ApoE ɛ4 carrier status.

ApoE ε4	Aβ40 mean ± SD (*n*)	Aβ42 mean ± SD (*n*)	t-Tau mean ± SD (*n*)
Non-carriers	58.59 ± 15.61* (125)	15.20 ± 2.17 (129)	18.90 ± 8.24 (129)
Carriers	52.36 ± 10.25* (31)	15.13 ± 2.02 (31)	20.83 ± 8.64 (31)

*Denotes statistical significance *p* < 0.05.

### Correlations of Age With the Concentrations and Ratios of Aβ40, Aβ42, and t-Tau

To determine the relationship between age and plasma concentrations and the ratios of Aβ40, Aβ42, and t-Tau, Spearman’s rank correlation analyses were performed. A significant positive correlation was detected between age and t-Tau concentrations (*r* = 0.127, *p* = 0.0120), and a negative correlation was detected between age and Aβ42 concentrations (*r* = −0.126, *p* = 0.0128). There was also a significant correlation with the ratio of Aβ42 to t-Tau (*r* = −0.155, *p* = 0.0022). These correlation coefficients were modest. No correlation was detected between age and Aβ40 concentrations or ratios.

### Relationship Between Core AD Bioarkers Within Age Groups

To further examine the age effect, we grouped all participants by 10-years intervals starting from age 20 to 29 years, the third decade. Aβ40, Aβ42, and t-Tau levels for these age groups are shown in Table 4. None of these biomarkers had age-group differences. To determine how age affected the relationship between the AD biomarkers in plasma, Spearman’s rank correlation analyses between the biomarkers within each age group were performed (Table 5).

**Table 4:** Plasma AD bioboconcentrations (pg/ml) by age groups.

Age groups	Aβ40 mean ± SD (*n*)	Aβ42 mean ± SD (*n*)	t-Tau mean ± SD (*n*)
20–29	62.21 ± 5.19 (21)	15.71 ± 0.41 (21)	16.51 ± 5.91 (21)
30–39	61.23 ± 9.14 (16)	15.57 ± 2.28 (16)	17.18 ± 6.12 (16)
40–49	56.48 ± 8.60 (15)	16.47 ± 3.73 (17)	18.83 ± 10.54 (16)
50–59	57.95 ± 11.30 (88)	15.42 ± 1.78 (93)	16.07 ± 7.92 (93)
60–69	59.58 ± 13.99 (132)	15.40 ± 2.52 (135)	17.85 ± 8.92 (135)
70–79	60.72 ± 15.02 (74)	15.13 ± 2.35 (77)	17.98 ± 9.21 (77)
80–91	58.61 ± 17.84 (32)	15.60 ± 2.23 (32)	22.37 ± 5.48 (32)

*SD, standard deviation; n, number of subjects*.

**Table 5:** Relationship between Aβ40, Aβ42, and t-Tau within each age group.

Age group	Aβ40 vs. t-Tau	Aβ42 vs. t-Tau	Aβ40 vs. Aβ42
20–29	*ρ* = −0.087	*ρ* = −0.308	*ρ* = −0.126
	*p* = 0.7074	*p* = 0.07074	*p* = 0.5875
	*n* = 21	*n* = 21	*n* = 21
30–39	*ρ* = 0.076	*ρ* = 0.282	*ρ* = −0.562
	*p* = 0.7783	*p* = 0.2893	*p* = 0.0235
	*n* = 16	*n* = 16	*n* = 16
40–49	*ρ* = −0.582	*ρ* = 0.444	*ρ* = −0.321
	*p* = 0.0228	*p* =0.0745	*p* = 0.2427
	*n* = 15	*n* = 17	*n* = 15
50–59	*ρ* = −0.445	*ρ* = 0.474	*ρ* = −0.261
	*p* < 0.0001	*p* < 0.0001	*p* = 0.0142
	*n* = 88	*n* = 93	*n* = 88
60–69	*ρ* = −0.458	*ρ* = 0.341	*ρ* = −0.303
	*p* < 0.0001	*p* < 0.0001	*p* = 0.0004
	*n* = 132	*n* = 135	*n* = 132
70–79	*ρ* = −0.293	*ρ* = 0.249	*ρ* = −0.116
	*p* = 0.0113	*p* = 0.0292	*p* = 0.3235
	*n* = 74	*n* = 77	*n* = 74
80–91	*ρ* = −0.252	*ρ* = 0.459	*ρ* = −0.120
	*p* = 0.1634	*p* = 0.0083	*p* = 0.5147
	*n* = 32	*n* = 32	*n* = 32

We observed an increasing presence of the relationship between AD biomarkers as age advances. A negative correlation was found between Aβ40 and Aβ42 in the age group ranging from 30 to 69 with the exception of the people in the 5th decade (age 40–49). Plasma t-Tau and Aβ40 concentrations were negatively correlated in age groups starting at age 40 and up to age 79. The t-Tau and Aβ42 concentrations correlated positively in age groups starting from age 50 to 59 to the oldest age group. These findings demonstrated that the levels of all three AD biomarkers in plasma became associated with each other in the 6th and 7th decades of life.

We also assessed whether the age group, ApoE ɛ4 carrier status, and plasma AD biomarkers could be useful for predicting the concentration of a specific AD biomarker (Table 6). Stepwise multiple regression analyses were performed for each biomarker by entering data in the order of age group, ApoE ɛ4 allele status, and the other two biomarkers. The results showed that ApoE ɛ4 allele status was not a significant contributor to the prediction of any biomarker levels. By contrast, age group contributed to the prediction of the AD biomarker concentrations. Plasma t-Tau concentrations could be predicted by a combination of age group and plasma concentrations of Aβ42 and Aβ40 with a coefficient of determination of 0.3701 (*p* < 0.0001). The coefficient of determination for predicting Aβ42 concentrations was 0.3433 (*p* < 0.0001) when age group and t-Tau concentrations were included in the model. The coefficient of determination for Aβ40 concentration prediction was 0.1205 (*p* = 0.0001) with age group and t-Tau in the model.

**Table 6:** Multiple regression of age group, ApoE ɛ4 allele, and plasma biomarkers.

	Variables in regression equation	*F*-ratios	*p*	Co-efficient of determination	Variables excluded from equation
Aβ40	Age group, t-Tau	7.13	= 0.0001	0.1588	ApoEɛ4, Aβ42
Aβ42	Age group, t-Tau	21.35	<0.0001	0.3612	ApoEɛ4, Aβ40
t-Tau	Age group, Aβ40, Aβ42	23.05	<0.0001	0.3702	ApoEɛ4

### Age-Related Reference Intervals of Plasma AD Biomarkers

**Figure 2:**
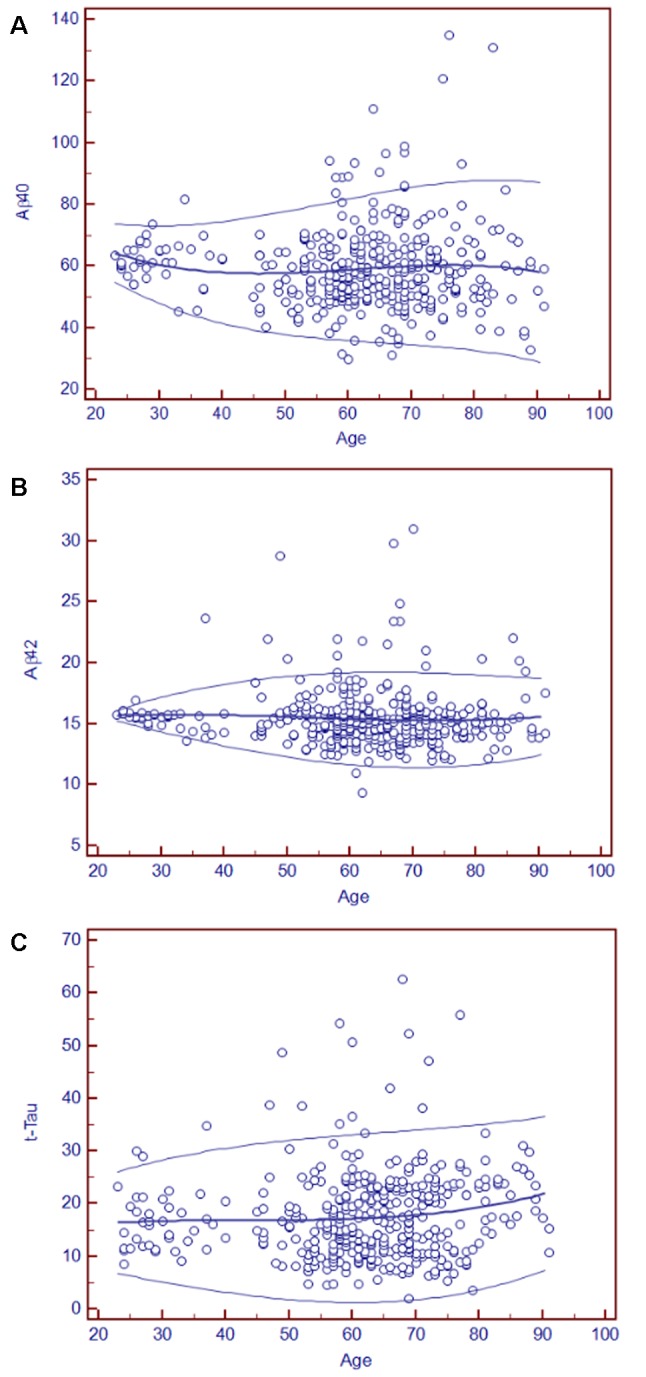
Age-related plasma biomarker reference intervals: means and centiles. The scatter plots illustrate the reference intervals for Aβ40 **(A)**, Aβ42 **(B)**, and t-Tau **(C)**. The age range in years is indicated on the x-axis, and the reference intervals of each marker are shown in circles in the figure. The units of the reference intervals are pg/ml. The central lines are the calculated means, and the top and bottom lines are the 90th and 10th centile curves.

As blood-based biomarkers have the potential to be used for screening subjects at the preclinical stage or those with MCI or early AD in the clinic or for clinical trials, we performed an analysis of the age-related reference intervals of Aβ40, Aβ42, and t-Tau concentrations from 391 subjects. The temporal concentration range of each biomarker is shown by age group at the 2.5th and 97.5th centiles in Table 7. Additional 10th and 90th centiles for each biomarker are illustrated in Figures 2A–C.

**Table 7:** Age-related plasma biomarker reference intervals (pg/ml).

Age (years)	Aβ40		Aβ42		t-Tau	
	0.025	0.975	0.025	0.975	0.025	0.975
30	47.87	72.98	14.35	17.18	5.18	28.33
40	41.37	74.42	13.19	18.26	3.23	30.56
50	37.73	77.74	12.27	18.90	1.83	32.06
60	35.80	81.82	11.65	19.18	1.24	33.11
70	34.48	85.52	11.42	19.20	1.77	34.01
80	32.62	87.71	11.65	19.02	3.69	35.02
90	29.0	87.28	12.43	18.72	7.29	36.45

## Discussion

In this cross-sectional study, we characterized the relationship between age and three plasma AD core biomarkers and the relationship between these biomarkers within a given age group. The 391cognitively normal subjects spanning 23–91 years of age were enrolled from 12 participating hospitals located in Asia, the USA, and Europe. The statistical analyses were performed in all participants as one large multicenter cohort. Our study detected weak but significant correlations between chronological years of age and plasma Aβ42 and t-Tau. Plasma Aβ42 decreased as age increased, in contrast to t-Tau, which increased with age. As the age span of the cohort was quite large, we grouped ages into 10-years intervals to assess whether the changes in plasma biomarker levels were more prominent in certain decades. By grouping ages into 10-years intervals, we did not detect age-group-associated differences in all three biomarkers. However, our important finding came from an analysis of the relationship between biomarkers within a given decade of life. There was an emerging prevalence of relationships between AD biomarkers during ages 50–70. Although no significant relationship between any two AD biomarkers was detected in the middle-aged groups, e.g., the 3rd and 4th decades of life, positive correlations between Aβ42 and t-Tau were detected from the 6th to 9th decades; negative correlations were detected between Aβ40 and Aβ42 in age groups from the 4th, 6th, and 7th decades and between Aβ40 and t-Tau during the 5th to 8th decades. During the 6th and 7th decade of life, all three biomarkers showed a significant relationship, positively correlated between Aβ42 and t-Tau and negatively correlated between Aβ40 and Aβ42 and Aβ40 and t-Tau. These relationships provided significant insights into the aging-associated patterns of changes in AD plasma biomarkers. Previous IMR studies reported AD-associated increases in Aβ42 and t-Tau (Chiu et al., 2013). This supports that during the 6th to 8th decades of life, the expression patterns of plasma biomarkers coincided with the pattern observed in MCI and early AD.

There have been only a few studies analyzing the effect of age on the levels of Aβ and t-Tau in incognitively normal subjects. One study reported a decrease in plasma Aβ40 levels with age (Kleinschmidt et al., 2016). Another study, dividing 245 subjects into age groups of young (≤34), adult (35≤ age ≤64), and old (>64), showed the lowest plasma Aβ42 concentration in the youngest group but no differences between the two older groups. The assay used in this study was Innogenetics ELISA (Belgium; Zecca et al., 2018). A study in Korean healthy adults aged 40–69 reported the effects of age and sex on the plasma levels and ratios of Aβ42 and Aβ40 (Kim et al., 2016). The assays of the study were also performed in ELISA format (Immuno-Biological Laboratories, Japan). A previous study on the plasma of healthy controls using IMR assays detected significantly higher tau levels in subjects aged 65–95 years than in the group aged 45–64 years (Chiu et al., 2017). If the participants in the present study were divided into only two groups, younger than 65 and older than 65, similar results were found (higher tau in the older group, *P* = 0.0378). The differences in findings between the reports could be due to the types of assays used for obtaining data, the age ranges, the age grouping, and the number of subjects in age groups.

The physiological significance of these relationships found in this study is not readily understood. It is possible that as age advances, various upstream/downstream molecular mechanisms in the brain (production, accumulation) and/or periphery (excretion, destruction) that affect circulating levels of Aβ40, Aβ42, and t-Tau could converge or interact, resulting in the formation of a relationship. Evidence has supported that amyloid pathological events in the brain precede and trigger Tau pathology and even synergize with each other (Small and Duff, 2008; Han and Shi, 2016). Notably, the relationships between these AD biomarkers were most prominently detected during the 6th and 7th decades of life, the time when AD pathology had increasingly accumulated in the brain and CSF Aβ42 changes were already detectable (Buchhave et al., 2012).

It has been demonstrated with different preclinical AD classification systems that in cognitively normal subjects in their 70s and older, AD pathology is prevalent (Dubois et al., 2016; Jack et al., 2016; Kern et al., 2018). Our finding that the relationship between plasma AD biomarkers was most prominently present in the age group of 60–69 suggested that age-related changes in the brain might be captured in plasma, as observed in the relationship between AD biomarkers during aging.

It has been well established that the decreases in CSF Aβ42 coincide with increased brain amyloid plaque pathology (Seeburger et al., 2015; Doecke et al., 2018). In CSF, Aβ changes were detected before the changes in t-Tau in cognitively normal adults (Buchhave et al., 2012), and the change could be observed as early as middle-age (Sutphen et al., 2015). It is important to establish the relationship of core AD biomarkers between CSF and plasma. A recent study reported a significant but small positive correlation between IMR-assayed plasma Aβ42 levels and ELISA-assayed CSF Aβ42 levels in incognitively normal subjects (Teunissen et al., 2018). Further studies using the same assay platforms are needed to understand this relationship.

The mechanisms by which the ApoE ɛ4 genotype increases the risk of developing AD have been an important topic of research. Much evidence has indicated that ApoE ɛ4 forms less stable complexes with Aβ (Chiu et al., 2013). How this might affect soluble Aβ levels in the circulation has not been understood because of the complexity of the potential mechanisms involved in brain and peripheral clearance as well as the degradation of the complexes. In this study, we detected higher plasma Aβ40 levels in ApoE ɛ4 noncarriers than in ApoE ɛ4 carriers. Nevertheless, a cross-sectional cognitively normal population-based study that determined how ApoE ɛ4 status affected the relationship between plasma levels of Aβ species and soluble receptors for Aβ, the soluble low-density lipoprotein receptor-related protein-1 (sLRP1) and soluble receptor for advanced glycation end products (sRAGE), found positive correlations of the receptors with Aβ40 but not with Aβ42 in ApoE ɛ4 noncarriers (Tai et al., 2014). This finding led to speculation that part of the reason why the noncarriers had higher levels of Aβ40 in plasma than the carriers could be because more clearance receptors were available in the noncarriers. This possibility will require further studies in different cohorts to validate the findings by Gao et al. (2018) and us as well as research to delineate the genotype-specific mechanisms in the brain-periphery clearance associated with these two Aβ receptors (Deane et al., 2009; Zlokovic et al., 2010).

## Conclusion

Previously, plasma levels of Aβ or t-Tau were considered to have limited value as biomarkers for disease classification. However, new technologies with superior technical sensitivity bring hope to the potential of using blood-based biomarkers in identifying preclinical, MCI, and early AD subjects. In this study, we provided the normal ranges of Aβ species and t-Tau in plasma as well as the development of a dynamic relationship between the biomarkers from middle to old age. Future studies will move towards a better understanding of the biology and dynamics of plasma Aβ and Tau in health and disease, as well as vigorous assessment of the clinical utility of IMR-assayed plasma Aβ and Tau in identifying preclinical AD, MCI, and early AD, and the study findings should be comparable with those from CSF biomarker and imaging studies.

## Data Availability

All datasets generated for this study are included in the manuscript.

## Ethics Statement

A total of 391 cognitively normal subjects aged 23–91 were enrolled from six hospitals in Taiwan: National Taiwan University Hospital (NTUH), Taipei Medical University Shuang-Ho Hospital (SHH), Renai Branch of Taipei City Hospital (RAH), Taipei Veterans General Hospital (TVUH), National Cheng Kung University Hospital (NCKUH), and Kaohsiung Chang Gung Memorial Hospital (KCCGMH); Sahlgrenska University (SU) in Guttenberg of Sweden; and Banner Sun Health Research Institute (BSHRI) in Sun City of Arizona of United States; two hospitals in the cities of Foshan and Guangzhou, China, and two clinics in Osaka of Japan from year 2010 to year 2018. Each participating hospitals and clinics followed the Institutional Research Board approved protocols for this research.

## Author Contributions

M-CP, T-FC, C-JH, L-KH, W-CL, C-CW, J-SJ, KB, MS, S-HY, P-NW, HH, SM, AT, YI, JL, HX, and M-JC enrolled subjects and performed clinical diagnosis for all participants. S-YY was responsible for IMR measurements. L-FL conducted the statistical analysis and prepared the manuscript. M-JC critically reviewed and revised the manuscript.

## Conflict of Interest Statement

S-YY is an employee of MagQu Company Limited and MagQu LLC. He is a shareholder of MagQu Company Limited. The remaining authors declare that the research was conducted in the absence of any commercial or financial relationships that could be construed as a potential conflict of interest.

## Footnotes

^1^www.medcalc.org
