# Corrigendum: Age-Dependent Relationship Between Plasma Aβ40 and Aβ42 and Total Tau Levels in Cognitively Normal Subjects

**DOI:** 10.3389/fnagi.2019.00292

**Published:** 2019-11-08

**Authors:** Lih-Fen Lue, Ming-Chyi Pai, Ta-Fu Chen, Chaur-Jong Hu, Li-Kai Huang, Wei-Che Lin, Chau-Chung Wu, Jian-Shing Jeng, Kaj Blennow, Marwan N. Sabbagh, Sui-Hing Yan, Pei-Ning Wang, Shieh-Yueh Yang, Hiroyuki Hatsuta, Satoru Morimoto, Akitoshi Takeda, Yoshiaki Itoh, Jun Liu, Haiqun Xie, Ming-Jang Chiu

**Affiliations:** ^1^Civin Neuropathology Laboratory, Banner Sun Health Research Institute, Sun City, AZ, United States; ^2^Division of Behavioral Neurology, Department of Neurology, National Cheng Kung University Hospital, College of Medicine, National Cheng Kung University, Tainan, Taiwan; ^3^Department of Neurology, National Taiwan University Hospital, College of Medicine, National Taiwan University, Taipei, Taiwan; ^4^Department of Neurology, Taipei Medical University, Taipei, Taiwan; ^5^Department of Neurology, Shuang Ho Hospital, Taipei Medical University, New Taipei City, Taiwan; ^6^Department of Neurology, Kaohsiung Chang Gung Memorial Hospital, Kaohsiung, Taiwan; ^7^Department of Internal Medicine, National Taiwan University Hospital, College of Medicine, National Taiwan University, Taipei, Taiwan; ^8^Clinical Neurochemistry Laboratory, Sahlgrenska University Hospital, Mölndal, Sweden; ^9^Department of Psychiatry and Neurochemistry, Institute of Neuroscience and Physiology, University of Gothenburg, Mölndal, Sweden; ^10^Lou Ruvo Center for Brain Health, Cleveland Clinic Nevada, Las Vegas, NV, United States; ^11^Department of Neurology, Renai Branch, Taipei City Hospital, Taipei, Taiwan; ^12^Department of Neurology, National Yang-Ming University, Taipei, Taiwan; ^13^Department of Neurology, Taipei Veterans General Hospital, Taipei, Taiwan; ^14^MagQu Company Limited, New Taipei City, Taiwan; ^15^MagQu LLC, Surprise, AZ, United States; ^16^Hatsuta Neurology Clinic, Osaka, Japan; ^17^Department of Neurology, Osaka City University Graduate School of Medicine, Osaka, Japan; ^18^Department of Physiology, School of Medicine, Keio University, Tokyo, Japan; ^19^Departemnt of Neurology, Sun Yat-Sen Memorial Hospital, Sun Yat-Sen University, Guangzhou, China; ^20^Department of Neurology, Foshan Hospital of Sun Yat-Sen University, Foshan, China

**Keywords:** Alzheimer, plasma, amyloid, tau, immunomagnetic reduction, cognitively normal subjects

In the original article, there was an error. The name of a participating site was incorrectly written as “Keio University Hospital.” The correct site is “Hatsuta Neurology Clinic.”

A correction has therefore been made to the table and legend for Table 1:

**Table 1:** The means and standard deviations (SD) of age (years) of the normal-cognition subjects in each participating site^*^.

**Site no**.	**Name of sites**	**Subject no**.	**Median (years)**	**Minimum (years)**	**Maximum (years)**	**Age, years Mean ± S.D**.
1	NTUH-1	90	57	23	81	53.60 ± 17.91
2	NTUH-2	79	69	56	89	70.35 ± 8.12
3	NTUH-3	24	46	26	89	49.54 ± 18.41
4	NCKUH	48	54	33	70	54.15 ± 7.49
5	SHH	38	65	56	76	64.97 ± 5.63
6	KCGMH	27	60	50	72	61.15 ± 4.93
7	SU	18	71	53	89	70.50 ± 9.60
8	BSHRI	16	82	71	91	81.94 ± 5.99
9	RAH	11	64	58	74	64.91 ± 5.05
10	TVGH	17	60	54	88	64.06 ± 10.23
11	SYSH	9	66	45	79	62.67 ± 10.25
12	HNC/ OCUH	14	65	53	83	64.93 ± 7.21

* *Normal cognition: CDR = 0, MMSE: 28–30, and meet NIA-AA guidelines published in 2011*. *Name of sites: NTUH, National Taiwan University Hospital; NCKUH, National Cheng Kung University Hospital; SHH, Shuang Ho Hospital; KCGMH, Kaohsiung Chang Gung Memorial Hospital; SUH, Sahlgrenska University Hospital; BSHRI, Banner Sun Health Research Institute; RAH, Renai Branch Taipei City Hospital; TVGHL, Taipei Veterans General Hospital; SYSMH, Sun Yet-Sen Memory Hospital; FH, Foshan Hospital; HNC, Hatsuta Neurology Clinic; OCUH, Osaka City University; SD, standard deviation*.

Additionally, a correction has also been made to the **Materials and Methods** section, subsection **Participating Sites**:

“A total of 391 cognitively normal subjects aged 23–91 were enrolled from 2010 to 2018 from the following six hospitals in Taiwan: National Taiwan University Hospital (NTUH), Taipei Medical University Shuang-Ho Hospital (SHH), Renai Branch of Taipei City Hospital (RAH), Taipei Veterans General Hospital (TVGH), National Cheng Kung University Hospital (NCKUH), and Kaohsiung Chang Gung Memorial Hospital (KCGMH); Sahlgrenska University Hospital (SUH) in Guttenberg, Sweden; Banner Sun Health Research Institute (BSHRI) in Sun City, AZ, USA; two hospitals in the cities of Foshan, Foshan Hospital (FH) and Guangzhou, Sun Yat-Sen Memorial Hospital (SYSMH), Guangdong, China; and finally two hospitals in Japan: Hatsuta Neurology Clinic (HNC) in Osaka, and Osaka City University Hospital (OCUH) in Osaka. All participants were older than 21 years of age and gave their own written informed consent. The study was approved by the Institutional Review Board (IRB) or Research Ethics Committee (REC) of each participating hospital in the respective countries, namely, NTUH REC, Taipei Medical University-Joint IRB for SHH, Taipei City Hospital REC for RAH, TVGH IRB, NCKUH IRB, KCGMH IRB, Central Ethical Review Board-University of Gothenburg for SUH, Banner Health IRB for BSHRI, Sun Yat-Sen University Hospital (SYSUH) Cancer Center IRB, Asai Dermatology Clinic IRB and Osaka City University IRB.”

And the subsection **Cognition Assessment and Criteria for Recruitment**:

“The purpose of the recruitment criteria was to exclude subjects with diagnoses of MCI and dementia. All study sites followed the NIA-AA criteria for the diagnosis of dementia and MCI due to AD (Albert et al., 2011; McKhann et al., 2011). In addition to clinical criteria, basic cognitive assessment tools [Mini-Mental State Examination (MMSE) and Clinical Dementia Rating (CDR)] were also used. The criteria for normal cognition were MMSE ≥ 28 and CDR = 0. Brain imaging and CSF biomarkers were used as supplementary tools. Brain (FDG)-PET were used by HNC/OCUH, Japan, and Subjects from SUH, Sweden had CSF Ab > 530 pg/ml and t-Tau <350 pg/ml (Sutphen et al., 2015; Teunissen et al., 2018). Subjects who had acute or chronic systemic diseases or neuropsychiatric disorders, visual or auditory dysfunction severe enough to interfere with cognitive assessments were all excluded.”

The authors apologize for these errors and state that this does not change the scientific conclusions of the article in any way. The original article has been updated.
